# Two *Anaplasma phagocytophilum* Strains in *Ixodes scapularis* Ticks, Canada

**DOI:** 10.3201/eid2012.140172

**Published:** 2014-12

**Authors:** Chantel N. Krakowetz, Antonia Dibernardo, L. Robbin Lindsay, Neil B. Chilton

**Affiliations:** University of Saskatchewan, Saskatoon, Saskatchewan, Canada (C.N. Krakowetz, N.B. Chilton);; Public Health Agency of Canada, Winnipeg, Manitoba, Canada (A. Dibernardo, L.R. Lindsay)

**Keywords:** *Anaplasma phagocytophilum*, human granulocytic anaplasmosis, gram-negative, bacteria, *Ixodes scapularis*, blacklegged ticks, PCR-RFLP, SNP, single nucleotide polymorphism, 16S rRNA gene, vectorborne, arachnid, Canada

## Abstract

We developed PCR-based assays to distinguish a human pathogenic strain of *Anaplasma phagocytophilum*, Ap-ha, from Ap-variant 1, a strain not associated with human infection. The assays were validated on *A. phagocytophilum*-infected blacklegged ticks (*Ixodes scapularis*) collected in Canada. The relative prevalence of these 2 strains in *I. scapularis* ticks differed among geographic regions.

The gram-negative bacterium *Anaplasma phagocytophilum* is the causative agent of human granulocytic anaplasmosis (HGA) in the United States (*1*). More than 90% of HGA cases occur in the Upper Midwest and Northeast (*2*). In these regions, blacklegged ticks (*Ixodes scapularis*) are the vectors of a human pathogenic strain (Ap-ha) and a variant strain (Ap-variant 1) of *A. phagocytophilum* (*3*–*7*), the latter of which appears not to be associated with human infection (*1*,*3*). HGA represents an emerging disease in southern Canada because populations of *I. scapularis* ticks have become established or are in the process of becoming established (*8*,*9*). However, there is limited information on the occurrence of *A. phagocytophilum* in these ticks (*10*–*12*) and the proportion of ticks infected with the Ap-ha strain.

The most commonly used method to distinguish the human pathogenic strain of *A. phagocytophilum* from those not associated with human infection is to sequence the 16S rRNA gene (*3*–*7*,*13*). The Ap-ha strain differs from the Ap-variant 1 strain by 2 nucleotide differences at the 5′ end of the gene sequence (*1*,*3*). However, there is a need for an alternative to currently used PCR-based methods for strain identification that are reliable, but faster and more cost-effective. Therefore, the objectives of the current study were to determine the proportion of blacklegged ticks infected with *A. phagocytophilum* in different geographic regions of Canada, and to develop restriction fragment length polymorphism (RFLP) and single nucleotide polymorphism (SNP) genotyping assays, targeting the 16S rRNA gene, to differentiate the Ap-ha strain from the Ap-variant 1 strain. We assessed the usefulness of these assays and determined the prevalence of the Ap-ha strain in *I. scapularis* ticks from different geographic regions of Canada.

## The Study

We conducted real-time PCR analyses targeting the *msp2* gene on genomic (g) DNA of 12,606 *I. scapularis* ticks collected across Canada (Technical Appendix 1). Of these, 169 (1.3%) ticks were PCR-positive (Table 1). There were significant differences (χ^2^_5_ = 129.7, p<0.001) in the proportions of ticks infected with *A. phagocytophilum* among geographic regions; with a greater proportion of PCR-positive ticks in Manitoba than in Ontario, Quebec, and the Atlantic provinces.

**Table 1:** *Anaplasma phagocytophilum*-PCR–positive blacklegged ticks collected from various provinces during 2007–2010, Canada

Location	2007			2008			2009			2010			2007–2010	
	No. ticks	No. +		No. ticks	No. +		No. ticks	No. +		No. ticks	No. +		No. ticks	No. + (%)
Prairie Provinces														
Alberta*	9	0		25	0		13	0		42	3		89	3 (3.4)
Saskatchewan*	5	2		3	1		1	1		1	0		10	4 (40.0)
Manitoba	35	2		156	4		119	6		260	20		570	32 (5.6)
Central Canada														
Ontario	1,187	4		1,402	3		856	3		962	3		4,407	13 (0.3)
Quebec	982	13		1,687	26		1,026	8		1,002	24		4,697	71 (1.5)
Atlantic Provinces														
New Brunswick	129	1		174	0		189	4		271	10		763	15 (2.0)
Nova Scotia	201	2		394	4		378	4		676	9		1,649	19 (1.2)
Prince Edward Island	54	3		76	1		107	3		122	3		359	10 (2.8)
Newfoundland*	11	0		9	0		15	1		27	1		62	2 (3.2)
Total	2,613	27		3,926	39		2,704	30		3,363	73		12,606	169 (1.3)

*Data from these provinces were not included in the statistical analyses because sample sizes (2007–2010) were <100 ticks.

**Figure 1:**
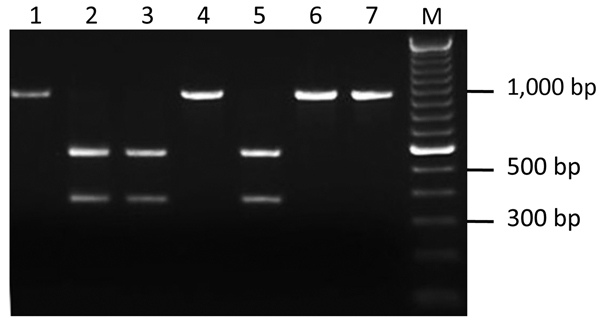
Restriction fragment length polymorphism patterns of 16S DNA for 7 *Anaplasma phagocytophilum* PCR–positive *Ixodes scapularis* ticks. All amplicons were produced by semi-nested PCR and digested with the restriction enzyme *Kpn2I*. Lane M, molecular mass marker.

We developed PCR-based assays to distinguish the Ap-ha strain from the Ap-variant 1 strain of *A. phagocytophilum* based on DNA sequence comparisons of the 16S rRNA gene of each strain (Technical Appendix 1) over a much larger region (875 bp) than in previous studies (*1*,*3*). In addition to the 2 nucleotide differences described previously (*1*,*3*), a third difference (at position 536) was detected in the aligned sequences (Technical Appendix 2). This nucleotide alteration in the DNA sequence of the Ap-variant 1 strain was associated with a restriction site for the endonuclease *Kpn2I* (T/CCGGA) that was absent in the sequence of the Ap-ha strain. Two PCR-RFLP assays (Technical Appendix 1), developed at different laboratories, were designed on the basis of this sequence difference and tested on 22 amplicons derived from *A. phagocytophilum*–infected ticks collected in Minnesota, USA (n = 17) and Manitoba (n = 5). Identical results were obtained for both assays. Three different RFLP patterns were produced. Eighteen amplicons remained undigested (i.e., a single band of ≈920 bp), and 3 amplicons had 2 bands (≈360 and ≈550 bp), representing the expected patterns for the Ap-ha strain and Ap-variant 1 strain, respectively (Figure 1). These results were confirmed on the basis of comparisons of the DNA sequences of representative samples. The RFLP pattern of 1 amplicon, derived from the gDNA of a female tick from Itasca State Park, Minnesota, consisted of 3 bands (≈360, ≈550 and ≈920 bp) (Technical Appendix 2), suggesting a mixture of the 2 strains. Additional analyses of another 125 amplicons from *A. phagocytophilum*-infected ticks revealed that 79 (63%) had RFLP patterns consistent with the Ap-variant 1 strain, and 46 (37%) had RFLP patterns of the Ap-ha strain. The DNA sequences of a subset of these samples (n = 58) showed 100% agreement between RFLP pattern and strain type of *A. phagocytophilum* (i.e., 24 of the Ap-ha strain and 34 of the Ap-variant 1 strain).

**Figure 2:**
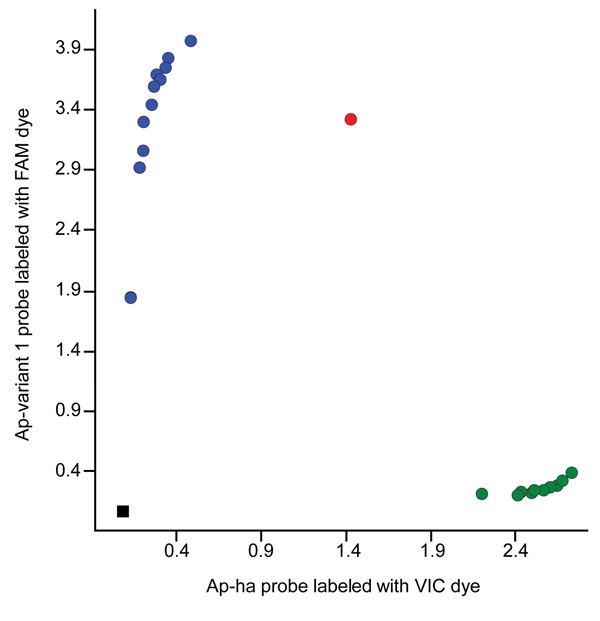
Allelic discrimination plot for the *Anaplasma* single nucleotide polymorphism assay based on the 16S RNA gene. Blue circles represent samples that contain the Ap-ha strain; green circles represent samples that contain the Ap-variant 1 strain. The red circle represents a sample containing a mixture of both strains. The black square represents the control (no template).

We also designed a custom TaqMan SNP genotyping assay (https://www.lifetechnologies.com/order/custom-genomic-products/tools/genotyping) to differentiate the two *A. phagocytophilum* strains on the basis of a nucleotide difference at the 5′ end of the 16S rRNA gene sequence (Technical Appendix 1). The SNP assay clearly discriminated between ticks infected with the Ap-ha and/or Ap-variant 1 strains (Figure 2). Of the 142 amplicons tested by this assay, 82 (58%) contained the Ap-variant 1 strain, 59 (42%) contained the Ap-ha strain, and 1 contained a mixture of both strains, which was in 100% agreement with the results of the RFLP analyses and DNA sequencing. The results of the SNP analyses also revealed a significant difference (χ^2^_2_ = 40.48, p<0.001) in the proportions of *I. scapularis* ticks infected with the Ap-ha strain among geographic regions (Table 2). A smaller proportion of ticks from Central Provinces were infected with the Ap-ha strain when compared with those from the Prairie and Atlantic Provinces.

**Table 2:** Black-legged ticks infected with the Ap-ha or Ap-variant 1 strain of *Anaplasma phagocytophilum* based on analyses using the TaqMan SNP genotyping assay,* Canada

Province	No. ticks	Ap-ha (%)	Ap-variant 1 (%)
Prairie Provinces			
Alberta†	1	1 (100)	0 (0)
Saskatchewan†	2	2 (100)	0 (0)
Manitoba	17	15 (88.2)	2 (11.8)
Central Canada			
Ontario	24†	2 (8.3)	22 (91.7)
Quebec	47	9 (19.1)	38 (80.9)
Atlantic Provinces			
Prince Edward Island	7	3 (42.9)	4 (57.1)
New Brunswick	10	6 (60.0)	4 (40.0)
Nova Scotia	17	8 (47.1)	9 (52.9)
Total	125	46 (36.8)	79 (63.2)

*http://www.lifetechnologies.com/search/global/searchAction.action?query=SNP&resultPage=1&resultsPerPage=15&autocomplete †Includes 13 ticks listed in Table 1 and an additional 11 *A. phagocytophilum*–infected ticks collected by drag sampling at 2 sites in Ontario.

## Conclusions

A small proportion (1.3%) of *I. scapularis* ticks collected in Canada were infected with *A. phagocytophilum*. The prevalence of *A. phagocytophilum–*-infected ticks differed among geographic regions, but the potential significance of this finding needs to be explored further. Although knowledge of the prevalence of *A. phagocytophilum–*infected *I. scapularis* ticks provides some information for determining the public health risks for HGA, the prevalence of the Ap-ha strain of *A. phagocytophilum* in blacklegged ticks should be considered for risk assessment, since the Ap-ha strain, and not the Ap-variant 1 strain, is most often associated with clinical cases of HGA (*4*).

For the current study, 3 PCR-based assays were developed to distinguish the Ap-ha strain from the Ap-variant 1 strain. DNA sequencing of representative samples confirmed the reliability of these assays. Each of the 3 assays detected the presence of both strains in the gDNA of a female tick. Mixed infections of both strains in *I. scapularis* ticks have been reported (*4*), but appear to be uncommon. Results of these assays also were 100% concordant with the results of 2 RFLP assays (developed at different laboratories) that were tested on the same gDNA samples. Similarly, there was total concordance in the identifications of the *A. phagocytophilum* strains present within 125 infected ticks collected across Canada by using the seminested PCR-RFLP assay and the TaqMan SNP genotyping assay. The results of these analyses revealed significant differences in the proportion of blacklegged ticks infected with the Ap-ha strain among geographic regions (p<0.001). The public health implications of these findings need to be examined in more detail, using the molecular assays developed in this study.

The TaqMan SNP genotyping assay is ideal for clinical and epidemiologic use where it may be essential to distinguish between the 2 strains of *A. phagocytophilum* in *I. scapularis* to assess the potential risk for human infection. However, in a clinical setting, it remains to be established how this assay would be incorporated into or supplement the current diagnostic approach for detecting *A. phagocytophilum* infections in humans. This test is less technically demanding and takes less time to perform than nested/seminested PCR-RFLPs and DNA sequencing analyses. Moreover, this method eliminates the need for postamplification manipulations and technical problems that are sometimes associated with RFLP analyses of amplicons produced by nested PCR (*14*,*15*). Nonetheless, given the 100% concordance in the results of the different analytical methods, the PCR-RFLP assays provide a reliable and cost-effective approach for distinguishing the Ap-ha strain from the Ap-variant 1 strain of *A. phagocytophilum*. The PCR-RFLP assays will be particularly useful in research laboratories that lack the capacity to conduct real-time PCRs providing an independent and relatively inexpensive method to confirm the results of the SNP assay.

Technical Appendix 1 Samples, DNA extraction and prevalence of *A. phagocytophilum* in *I. scapularis*

Technical Appendix 2 Genetic test results for *A. phagocytophilum*
