# Supplementary material for: Two Anaplasma phagocytophilum Strains in Ixodes scapularis Ticks, Canada
Source: Emerg Infect Dis. 2014 Dec;20(12):2064–7. doi: 10.3201/eid2012.140172 (PMC4257797; doi:10.3201/eid2012.140172)
Supplement: Technical Appendix 1 — Samples, DNA extraction and prevalence of A. phagocytophilum in I. scapularis [file 14-0172-Techapp-s1.pdf]

DOI: http://dx.doi.org/10.3201/eid2012.14012

Two *Anaplasma phagocytophilum* Strains in *Ixodes scapularis* Ticks, Canada

Technical Appendix 1. Samples, DNA extraction and prevalence of *A. phagocytophilum* in *I. scapularis*

Samples, DNA extraction and prevalence of *A. phagocytophilum* in *I. scapularis*

Blacklegged ticks (*n*=12,606) were collected over four years (2007-2010) as part of a national passive tick surveillance program conducted in Canada by the National Microbiology Laboratory (NML) using the methods described previously (*1*). Adult *I. scapularis* were also collected by drag sampling: in 2008 from Itasca State Park, Camp Ripley and St. Croix State Park (*n*=56 per locality) in Minnesota (USA), in 2010 from within Pembina Valley Provincial Park (*n*=46), and along the Stanley Trail (*n*=44), in southern Manitoba, and in 2009 from the Wainfleet Bog Conservation area and St. Lawrence Islands National Park in southern Ontario (*n*=11). Total genomic (g) DNA was extracted from each tick at the NML or the University of Saskatchewan (U of S) using either the QIAamp DNA Mini Kit or DNeasy 96 Blood and Tissue Kit (QIAGEN, Ontario, Canada) as per the manufacturer's instructions, but with the modifications described previously (*1*,*2*). The gDNA of *A. phagocytophilum* was also extracted (*1*) from an equine isolate (MN-93) propagated in the HL-60 promyelocytic cell line (ATCC CCL-240). This sample was used as a positive control in PCR assays conducted at the NML.

The gDNA of all 12,606 *I. scapularis* from the passive surveillance program were screened by real-time PCR targeting the *msp2* gene to determine if they were infected with *A. phagocytophilum*. Real-time PCR was conducted using a 7500 or 7900HT SDS Real-time PCR System (Applied Biosystems; California, USA), and the conditions described by Courtney et al. (*3*). All PCR-positive samples were confirmed using a real-time PCR targeting the 16S rRNA gene of *A. phagocytophilum* (*4*). Contingency (χ^2) tests were used to determine if the relative number (i.e. percentage) of *A. phagocytophilum*-infected ticks differed among geographical regions.

Comparison of the 16S rRNA sequences of the Ap-ha and Ap-variant 1 strains

The DNA sequences of a part (870 bp) of the 16S rRNA gene were determined for *A. phagocytophilum* present in *I. scapularis* adults collected from Minnesota. These tick samples were selected because both the Ap-variant 1 and Ap-ha strains have been reported in *I. scapularis* from the Upper Midwest of the United States (*5*). Initially, the presence/absence of *A. phagocytophilum* in each tick was determined by targeting part of the bacterial 16S gene using nested (n) PCR. Primers EC12 (5'-TGATCCTGGCTCAGAACGAACG-3') and EC9 (5'-TACCTTGTTACGACTT-3') (*6*) were used in phase 1, while primers HGE1F (5'-GGATTATTCTTTATAGCTTGCT-3') and HGE3R (5'-TTCCGTTAAGAAGGATCTAATCTC-3') (*7*) were used phase 2 of the nPCR. All PCRs were conducted in 25 µl reaction volumes containing 3 mM $MgCl_2$, 200 µM of each dNTP, 25 pmol of each primer, 0.5 U of *Taq* DNA polymerase (Bio-Rad, Ontario, Canada), and 1 µl of gDNA (for phase 1) or 1 µl of purified amplicon (for phase 2). Amplicons (10 µl) were purified by adding exonuclease I (3 U) (New England BioLabs, Ontario, Canada) and shrimp alkaline phosphatase (0.15 U) (Fermentas, Ontario, Canada) and PCR buffer (1 µl) prior to incubation at 37°C for 15 min, and 80°C for 15 min. PCRs were performed using the following conditions; for phase 1: 95°C for 5 min, followed by 30 cycles of 95°C for 60 s, 58°C for 60 s, and 72°C for 60 s, and a final extension at 72°C for 5 min, and for phase 2: 95°C for 5 min, then 30 cycles of 94°C for 45 s, 60°C for 45 s, and 72°C for 45 s, and a final extension at 72°C for 5 min. Negative (i.e., no gDNA) controls were included in each set of reactions. The amplicons of seven of 17 PCR-positive samples were purified (as described above) and sequenced using primers HGE1F and HGE3R (in separate reactions). Sequencing was conducted at the National Research Council (Saskatoon) on an ABI 3730xl capillary sequencer using BigDye™ Terminator version 3.1 cycle sequencing kits (Applied Biosystems). The sequences of the two strains have been deposited in GenBank under the accession numbers HG916766 and HG916767. Sequence data were compared to the 16S rRNA sequences of *A. phagocytophilum* on GenBank using BLAST.

The analyses of the Blast searches revealed that the DNA sequences of five amplicons were 100% identical to the sequence of the 16S rRNA gene of the Ap-ha strain (accession no. U02521), whereas those of the other two amplicons differed at three nucleotide positions (alignment positions 3, 11, and 536) compared to the sequence of the Ap-ha strain (Table 1). The first 375 bp of these two amplicons were identical in sequence to the 16S rDNA sequence of the

Ap-variant 1 strain (accession no. AY193887); however, a comparison over the 870 bp was not possible since there is no sequence data available for this part of the gene for the Ap-variant 1 strain.

PCR-RFLP

Data analyses using the program Webcutter 2.0 (http://rna.lundberg.gu.se/cutter2) revealed that the nucleotide alteration in the DNA sequence of the Ap-variant 1 strain was associated with a restriction site for the endonuclease *Kpn2I* (T/CCGGA) that was absent in the sequence of the Ap-ha strain. A nested PCR-RFLP assay, developed (at the U of S) based upon this restriction site was tested on the *A. phagocytophilum*-positive amplicons derived from the ticks collected in Minnesota. RFLP digests were performed in 30 µl volumes containing 15.5 µl of nuclease free H_2O, 2.5 µl of the restriction enzyme *Kpn2I* (FastDigest®, Fermentas), 2 µl of 10x FastDigest® Green Buffer and 10 µl of unpurified PCR product from the second phase of the nested PCR (described above). Digests were performed at 37°C for 2 hrs and then at 80°C for 15 mins prior to loading (10 µl of product) on 1% agarose-TBE gels that were subjected to electrophoresis (100 volts for 2 hrs). A nested PCR was then conducted on the total gDNA of the 90 ticks collected from southern Manitoba, and the RFLP assay applied to all PCR-positive samples. The undigested products of three PCR-positive samples were purified and sequenced using primers HGE1F and HGE3R (in separate reactions) to confirm that there was 100% concordance between RFLP patterns and stain type of *A. phagocytophilum*.

A second PCR-RFLP assay was developed and tested (at NML) using amplicons of the bacterial 16S gene produced by semi-nested PCR. This modification was introduced because the semi-nested PCR produced better quality amplicons from a few gDNA samples compared to those produced by nested PCR. In the first phase of the semi-nested PCR, part (932 bp) of the 16S gene was amplified using primers Ge3a (5’- CACATGCAAGTCGAACGGATTATTC-3’) and Ge10r (5’- TTCCGTTAAGAAGGATCTAATCTCC-3’) and the cycling conditions of Massung et al. (*8*). Then, a smaller fragment (919 bp) was amplified using primers Ge9f (5’-AACGGATTATTCTTTATAGCTTGCT-3’) (*8*) and Ge10r, and the same cycling conditions as for phase 1, except that 30 cycles were used, and the initial denaturation period was 5 min. PCRs were conducted in 50 µl reaction volumes containing 3 mM $MgCl_2$, 200 µM of each dNTP, 0.2 µM of each primer, 2.5 U of i*Taq* DNA polymerase (Bio-Rad), and 3 µl of gDNA (for phase 1)

or 2 µl of unpurified amplicon (for phase 2). The 919 bp amplicons were digested with *Kpn2I* as described above. This PCR-RFLP assay was tested on the gDNA of 114 of the 169 ticks from the passive surveillance program that were positive for *A. phagocytophilum* (i.e., by *msp2* and 16S rRNA gene real-time PCRs) and 11 *A. phagocytophilum*-infected ticks collected by drag sampling at two sites in Ontario. These 125 gDNA samples were selected based on their cycle threshold (Ct) value in the *msp2* real-time PCR assay; all samples had Ct values ≤ 35. A subset (*n*=58) of undigested amplicons were purified using Montage filter units (Millipore) and sequenced using primers Ge9f and Ge10r to confirm 100% concordance between RFLP pattern and strain type of *A. phagocytophilum*. The gDNA of all *A. phagocytophilum*-positive ticks from Minnesota previously tested by nested PCR-RFLP at U of S were also subjected to the semi-nested PCR-RFLP assay (at NML) to test for concordance. Sequencing was conducted on an ABI 3130xl Genetic Analyzer using BigDye™ Terminator version 3.1 cycle sequencing kits (Applied Biosystems). Sequence data was analyzed using DNASTAR Lasergene 9 Software and compared to those on GenBank using BLAST.

SNP assay

A custom TaqMan® SNP Genotyping Assay (Applied Biosystems, California, USA) was designed to determine the *A. phagocytophilum* strains present in the gDNA of 125 *I. scapularis* from the passive surveillance program. These representative samples were all confirmed to be positive for *A. phagocytophilum* by real-time PCR (*msp2* and the 16S gene), and typed as either Ap-ha or Ap-variant 1 strain by PCR-RFLP. They also covered the geographical range of the tick submissions, different collection years, and a range of Ct values. The 17 *A. phagocytophilum*-infected ticks from Minnesota were also used to validate the SNP assay. The SNP Genotyping Assay mix contained two primers (Ap Forward: 5’-ACATGCAAGTCGAACGGATTATTCT-3’ and Ap Reverse: 5’- GCTATCCCATACTACTAGGTAGATTCCT-3’) that flank the region (50 bp) containing two SNP sites (alignment positions 3 and 11; Table 2), and two TaqMan probes, one that matched the Ap-ha strain (Ap-ha probe: 5’-CTGCCACTAACTATTCT-3’) labelled with VIC, the other designed for the Ap-variant 1 strain (Ap-var 1 probe: 5’-CTGCCACTAATTATTCT-3’) labelled with 6-carboxy-fluorescein (FAM) and a non-fluorescent quencher with a minor groove binder at the 3’ end. Reaction mixtures (25 µl) contained 1.25 µl of 20X Custom TaqMan® SNP Genotyping Assay, 12.5 µl of TaqMan Universal Master Mix (Applied Biosystems), 6.25 µl nuclease-free water and 5 µl of gDNA (or

water for the negative controls). Real-time PCRs were performed in a 7500 Real-time PCR System (Applied Biosystems) using the following amplification conditions; 50°C for 2 min, 95°C for 10 min, and then 40 cycles of 95°C for 15 s and 60°C for 1 min. Strain assignment was performed using the AB 7500 version 2.0.5 software. A strain discrimination plot was generated using the R_N (fluorescence) from the Ap-ha probe versus the R_N from the Ap-var 1 probe. Contingency (χ^2) tests were used to determine if the proportions of ticks infected with the Ap-ha and Ap-variant 1 strains differed among geographical regions.

Extracts containing DNA from *Borrelia burgdorferi*, *B. miyamotoi*, *Ehrlichia chaffeensis* and *Babesia microti* were used to test the specificity of the SNP assay. In each case, detectable amplification products were not produced for further genotyping. In order to determine the limit of detection, serial dilutions of DNA from *A. phagocytophilum* propagated in HL-60 cells were used to generate a standard curve. The dynamic range of the assay spanned from 90 ng/µl DNA to 28 pg/µl DNA which corresponded to Ct values of 28 and 37, respectively.

References

1. Ogden NH, Trudel L, Artsob H, Barker IK, Beauchamp G, Charron DF, et al. *Ixodes scapularis* ticks collected by passive surveillance in Canada: analysis of geographic distribution and infection with Lyme borreliosis agent *Borrelia burgdorferi.* J Med Entomol. 2006;43:600–9. PubMed http://dx.doi.org/10.1603/0022-2585(2006)43[600:ISTCBP]2.0.CO;2

2. Dergousoff SJ, Chilton NB. Differentiation of three species of ixodid tick, *Dermacentor andersoni, D. variabilis* and *D. albipictus*, by PCR-based approaches using markers in ribosomal DNA. Mol Cell Probes. 2007;21:343–8. PubMed http://dx.doi.org/10.1016/j.mcp.2007.04.003

3. Courtney JW, Kostelnik LM, Zeidner NS, Massung RF. Multiplex real-time PCR for detection of *Anaplasma phagocytophilum* and *Borrelia burgdorferi.* J Clin Microbiol. 2004;42:3164–8. PubMed http://dx.doi.org/10.1128/JCM.42.7.3164-3168.2004

4. Dibernardo A, Cote T, Ogden NH, Lindsay RL. The prevalence of *Borrelia miyamotoi* infection, and co-infections with other *Borrelia* spp. in *Ixodes scapularis* ticks collected in Canada. Parasites & Vectors. 2014;7:183. http://dx.doi.org/10.1186/1756-3305-7-183

5. Michalski M, Rosenfield C, Erickson M, Selle R, Bates K, Essar D, et al. *Anaplasma phagocytophilum* in central and western Wisconsin: a molecular survey. Parasitol Res. 2006;99:694–9. PubMed http://dx.doi.org/10.1007/s00436-006-0217-9

6. Kawahara M, Rikihisa Y, Lin Q, Isoagai E, Tahara K, Itagaki A, et al. Novel genetic variants of *Anaplasma phagocytophilum*, *Anaplasma bovis*, *Anaplasma centrale*, and a novel *Ehrlichia* sp. in wild deer and ticks on two major islands in Japan. Appl Environ Microbiol. 2006;72:1102–9. PubMed http://dx.doi.org/10.1128/AEM.72.2.1102-1109.2006

7. Adelson ME, Rao R-VS, Tilton RC, Cabets K, Eskow E, Fein L, et al. Prevalence of *Borrelia burgdorferi*, *Bartonella* spp., *Babesia microti*, and *Anaplasma phagocytophila* in *Ixodes scapularis* ticks collected in northern New Jersey. J Clin Microbiol. 2004;42:2799–801. PubMed http://dx.doi.org/10.1128/JCM.42.6.2799-2801.2004

8. Massung RF, Slater K, Owens JH, Nicholson WL, Mather TN, Solberg VB, et al. Nested PCR assay for detection of granulocytic Ehrlichiae. J Clin Microbiol. 1998;36:1090–5. PubMed
